# Metabolic Insight into Cold Stress Response in Two Contrasting Maize Lines

**DOI:** 10.3390/life12020282

**Published:** 2022-02-14

**Authors:** Tao Yu, Jianguo Zhang, Jingsheng Cao, Xin Li, Sinan Li, Changhua Liu, Lishan Wang

**Affiliations:** 1 Maize Research Institute of Heilongjiang Academy of Agricultural Sciences, Harbin 150086, China; hljnkyyms@163.com (T.Y.); maize_lee@163.com (X.L.); mrlee890323@163.com (S.L.); 2 College of Advanced Agriculture and Ecological Environment, Heilongjiang University, Harbin 150080, China; liuchanghua70@163.com (C.L.); wdl971108@163.com (L.W.)

**Keywords:** abiotic stress, cold stress, maize, metabolites, flavonoids, crop improvement

## Abstract

Maize (*Zea mays* L.) is sensitive to a minor decrease in temperature at early growth stages, resulting in deteriorated growth at later stages. Although there are significant variations in maize germplasm in response to cold stress, the metabolic responses as stress tolerance mechanisms are largely unknown. Therefore, this study aimed at providing insight into the metabolic responses under cold stress at the early growth stages of maize. Two inbred lines, tolerant (B144) and susceptible (Q319), were subjected to cold stress at the seedling stage, and their corresponding metabolic profiles were explored. The study identified differentially accumulated metabolites in both cultivars in response to induced cold stress with nine core conserved cold-responsive metabolites. Guanosine 3′,5′-cyclic monophosphate was detected as a potential biomarker metabolite to differentiate cold tolerant and sensitive maize genotypes. Furthermore, Quercetin-3-O-(2″′-p-coumaroyl)sophoroside-7-O-glucoside, Phloretin, Phloretin-2′-O-glucoside, Naringenin-7-O-Rutinoside, L-Lysine, L-phenylalanine, L-Glutamine, Sinapyl alcohol, and Feruloyltartaric acid were regulated explicitly in B144 and could be important cold-tolerance metabolites. These results increase our understanding of cold-mediated metabolic responses in maize that can be further utilized to enhance cold tolerance in this significant crop.

## 1. Introduction

The temperature threshold for optimum growth during the development process has gained much attention during the past few decades as most of the crops are being cultivated outside their optimal growth environments to meet food supply demand [1]. Maize is mainly considered a cold-sensitive crop, with a variable threshold for different growth stages [2]. Early growth stages have a relatively lower threshold (5–18 °C) for optimal growth compared to later growth stages (25–28 °C) [3,4]. Although the lowest acceptable temperature for germination is considered 5 °C, a decrease in temperature below 10 °C delayed the emergence and germination process resulting in an increased growth period and reduced yield [3]. Generally, early sowing is recommended for a maize crop to avoid high temperatures during the late growth stages [5]. Therefore, chilling susceptibility in maize crop is one of the major concerns for optimum yield, which is drastically reduced when the plant is subjected to early-stage cold stress [6,7]. Although much work has been done to understand the biological mechanisms behind cold tolerance [8,9,10], there is a lack of studies addressing the metabolic responses resulting in increased/decreased tolerance levels in maize. 

Self-defense as plant response towards biotic or abiotic stress is mainly a set of mechanisms controlled by modified gene expression resulting in differential protein synthesis affecting metabolic pathways [11]. Responses leading towards cold tolerance can be primarily attributed to the structural and functional modification of plasma membrane, synthesis and activation of cryoprotectant molecules, and enrichment of reactive oxygen species [12]. Moreover, cold-responsive mechanisms are interlinked through signaling pathways, such as MAPK signaling and plant hormone signaling, governed by changes in hormonal levels [13]. Advances in metabolomics have enabled us to understand better these responses and deviations in metabolic pathways [14], as metabolome is at the receiving end of biological information [15]. Considerable variation concerning cold stress is present in the maize germplasm worldwide [16,17,18]. Previous reports suggested a significant reduction in photosynthesis under cold tolerance [1,19,20,21], resulting in reactive oxygen species (ROS) production [22]. One of the major protective mechanisms against ROS accumulation in maize is the translocation of metabolites in leaves [23,24,25]. Another report suggested a positive regulation of amino acid metabolism accompanied by fatty acid metabolism with Carnitine up-accumulation [26]. Furthermore, our recent report emphasized the enrichment of phenylpropanoid biosynthesis pathways and phenolic biosynthesis pathways under cold stress [27]. The cold regulatory gene network contributes to developing resistance towards cold tolerance in plants [28]. Despite some breakthroughs in understanding the genetic mechanisms behind cold tolerance, much remains ambiguous in complex regulatory networks coping with cold tolerance. 

Recent advances in omics have enabled us to decipher complex regulatory networks about plants’ stress resistance, specifically cold tolerance [28,29,30,31]. Through an integrated approach, previously published reports characterized multiple metabolite synthesis pathways, i.e., glucosinolate metabolism [29] and flavonoid biosynthesis [8,30] in Arabidopsis, anthocyanin biosynthesis in maize [23], and combined abiotic stress responses (CAbS) in rice [32]. However, cold tolerance mechanisms remain largely unclear in maize regarding the metabolome. Therefore, this study aimed at deciphering the metabolome changes occurring as a response to cold stress in two contrasting maize lines. 

## 2. Materials and Methods

### 2.1. Plant Materials

This study used two inbred maize lines B144 (tolerant) and Q319 (sensitive). These lines were selected based on a previously conducted screening study for cold tolerance. Maize Research Institute of Heilongjiang Academy of Agricultural Sciences, China, provided the seeds for the test material. B144 is a highly tolerant maize inbred line, while Q319 is highly susceptible to cold tolerance [27]. Each inbred line was sown in pots in 5 replicates, each with ten plants. Before sowing, 0.5% sodium hypochlorite solution was used to surface-sterilize the seeds, followed by washing with distilled water. After germination, pots were kept in an incubation chamber (GEN1000, Conviron, USA) with environmental parameters as temperature; 25 °C and photoperiod; 12/12 h (12 h light followed by 12 h dark). Plants with uniform germination times were observed for ten consecutive days. After ten days, at BBCH scale 12 with two leaves unfolded [33], the temperature was dropped to 5 °C to induce a cold stress response. While under control conditions, the temperature and photoperiod were kept constant. The leaf samples were collected for further analysis from test material after 24 h. The samples were collected with three biological and technical repeats. All the samples were stored at −80 °C until further process. 

### 2.2. Sample Preparation

The leaf samples from each line were prepared following Deng et al. [34]. The collected samples were cryopreserved and freeze-dried in a vacuum and then ground to powder. One hundred micrograms of ground powder were weighed, followed by thawing in 1.2 mL methanol extract (Shanghai Aladdin Bio-Chem Technology Co., Ltd., Shanghai, China) for each sample. After cyclic centrifugation (30 s after every 30 min), the samples were kept at 4 °C overnight. The samples were centrifuged again at 12,000 rpm for 10 min, and the supernatant was collected. Filtered samples were sorted for further analysis.

### 2.3. UPLC-MS/MS Analysis

In this study, metabolome as a cold stress response in two inbred maize lines was quantified using ultra-performance liquid chromatography-tandem mass spectrometry (UPLC-MS/MS). UPLC-MS/MS was performed by Metware, Wuhan, China, on a UPLC-MS/MS system (UPLC, SHIMADZU NexeraX2, www.shimadzu.com.cn/; accessed on 11 November 2021; MS, Applied Biosystems 4500 Q TRAP, www.appliedbiosystems.com.cn/, accessed on 11 November 2021). The analytical conditions were kept as elaborated by Gao et al. [35]. In brief, UPLC: column, Agilent SB-C18 (1.8 µm, 2.1 mm × 100 mm) was used while the mobile phase with A (pure water with formic acid) and B (acetonitrile with formic acid) solvents was standardized. The gradient program was used starting from 95% A and 5% B for 9 min and then changed to 5% A and 95% B for 1 min. The gradient was then adjusted back to 95% A and 5% B. The flow velocity was adjusted to 0.35 mL/min throughout the gradient process with a constant temperature of 40 °C and 4 μL injection volume.

Furthermore, a triple quadrupole-linear ion trap mass spectrometer (AB4500 Q TRAP UPLC/MS/MS) system with ESI turbo Ion spray interface was employed for quadrupole-linear ion trap (Q TRAP-MS/MS). To control the system parameters, Analyst 1.6.3 was used [36]. The ESI source operation parameters were kept as explained by [37].

### 2.4. Quality Control and Data Analysis

Quality check for metabolome datasets was performed to verify the reproducibility following the description of Fiehn et al. [30]. Mixed samples were added to each sample, and changes were monitored. Analyst 1.6.3 software (AB SCIEX, Ontario, ON, Canada) was employed to obtain descriptive statistics for each metabolome dataset. Further, principal component analysis (PCA) was performed to check the variability in the datasets. Unconstrained PCA was performed using statistics function prcomp within R (www.r-project.org, accessed on 11 November 2021). Data sets were grouped based on PCs viz., PC1, and PC2. Moreover, the correlation between different metabolome datasets was estimated using R.

### 2.5. Identification of Differential Metabolites

The differential accumulation of metabolites (DAMs) between two inbred maize lines under cold stress compared with the control conditions was estimated using variable importance in projection (VIP). The criteria for DAMs was set as VIP > 1 and Log2 fold change > 1(log2FC > 1). Orthogonal Projections to Latent Structures-Discriminant Analysis (OPLS-DA) was estimated using Log2FC followed by mean centering. VIP scores were obtained from OPLS-DA estimates, R software with MetaboAnalystR package was used for Log2FC, VIP, and OPLS-DA estimations.

## 3. Results

### 3.1. Metabolome Profiling in Two Contrasting Maize Lines under Cold Stress Treatment

The metabolic changes associated with cold tolerance in two maize inbred lines (B144; cold-tolerant and Q319; cold-susceptible) were studied using leaf samples from control and cold stressed plants based on the mass spectrography (UPLC-MS/MS) technology. With constant quality checks by observing the accuracy of the instrument, the repeatability of metabolite detection was confirmed, as previously explained by Fiehn et al. [38]. In quality control samples, the total ion current depicted consistent and overlapped peaks, suggesting the reliability of the observations (Figure S1).

**Figure 1:**
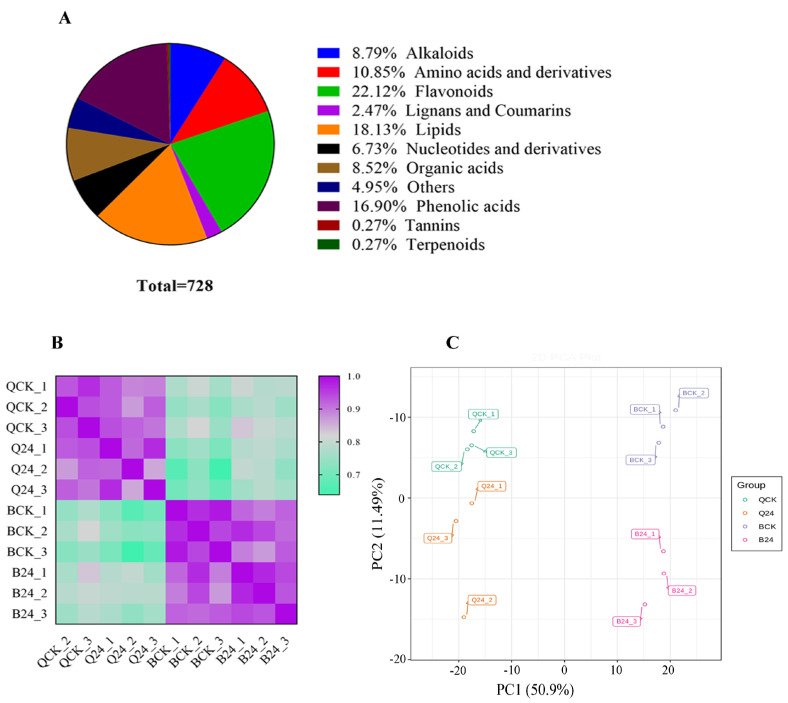
Metabolome quality control and description (**A**) Major classes of identified metabolites (728) and their corresponding percentage (**B**) correlation matrix for metabolites identified in leaf tissues of B144 and Q319 inbred maize lines (**C**) Principal component analysis for metabolites identified in leaf tissues of B144 and Q319 inbred maize lines. * QCK: Q319 control; Q24: Q319 cold stress; BCK: B144 control; B24: B144 cold stress.

As a result of UPLC-MS/MS, we identified 728 metabolites (Table S1). Owing to the metabolite structures, the identified metabolites were categorized into eleven major classes (Figure 1A), including flavonoids (22.12%), lipids (18.13%), phenolic acids (16.90%), amino acids (10.85%), alkaloids (8.79%), organic acids (8.52%), nucleotides and derivatives (6.73%), lignans and coumarins (2.47%), tannins (0.2%), terpenoids (0.2%), and others (4.95%). Comprehensive information about the set of identified metabolites, including molecular weights (Da), compound formula, ionization, compounds, classes, and KEGG pathways, are listed in Table S1.

Mass spectrography results were further verified, and we performed a correlation analysis and a principal component analysis (PCA) for the samples based on their corresponding ion intensity values (Figure 1B,C). Correlation results suggested a strong correlation among samples from the same genotype, indicating the reliability of metabolome data. Moreover, the PCA classified samples into four groups, and replications from each group were grouped together. PCA-estimates covered 62.39% variation, with PC1 accounting for 50.9% variability and PC2 covered 11.49% variation. PCA results verified the reliability of the metabolome dataset.

### 3.2. Cold Stress-Mediated Metabolic Responses in Two Inbred Maize Lines

**Figure 2:**
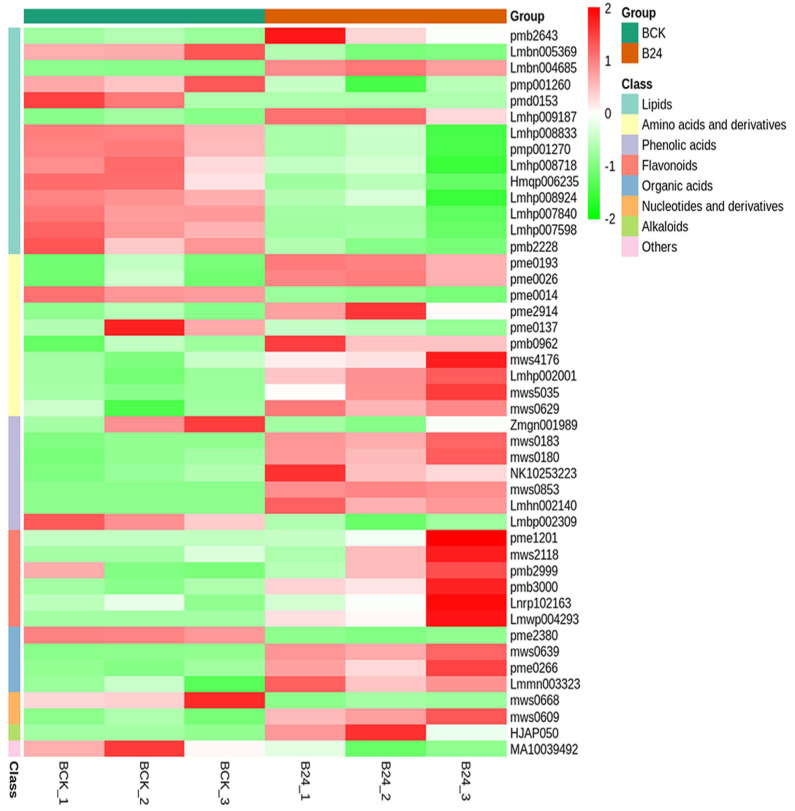
Differential accumulation of metabolites in B144 (cold tolerant maize inbred line). Heatmap representing accumulation profile of DAMs under control (BCK) and cold-stress (B24).

**Figure 3:**
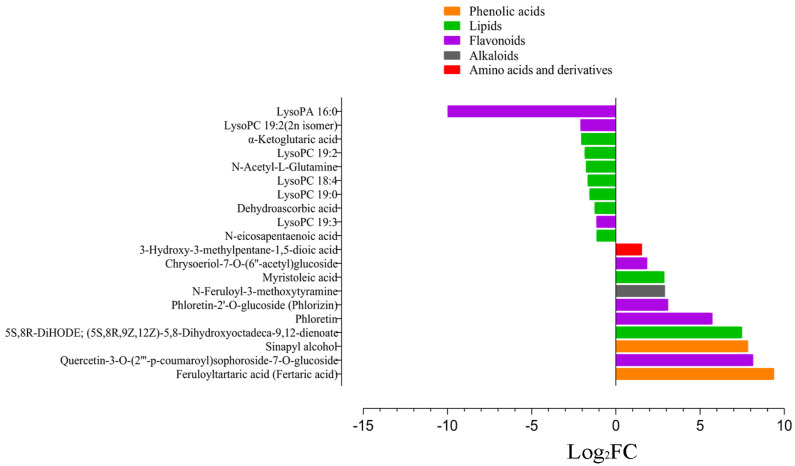
Top metabolites differentially accumulated between BCK and B24, where the x-axis represents log2FC of ion intensity. * BCK: B144 control; B24: B144 cold stress.

Maize is a thermophilic plant; therefore, it is pertinent to understand and exploit changes associated with cold stress at the early growth stage. To serve the purpose, we exposed a cold-tolerant maize inbred line B144 to cold stress and compared its metabolome with the control. Comparing the metabolites ion intensity in the tolerant line between control and stress conditions yielded a total of 45 differentially accumulated metabolites (DAM) (Figure 2 and Table S2). Among these DAMs, 27 metabolites were up-accumulated, while 18 metabolites were down-accumulated under cold stress. Feruloyltartaric acid (Fertaric acid), Quercetin-3-O-(2″′-p-coumaroyl)sophoroside-7-O-glucoside, Sinapyl alcohol, 5S,8R-DiHODE; (5S,8R,9Z,12Z)-5,8-Dihydroxyoctadeca-9,12-dienoate, Phloretin, Phloretin-2′-O-glucoside (Phlorizin), N-Feruloyl-3-methoxytyramine, Myristoleic acid, Chrysoeriol-7-O-(6″-acetyl)glucoside, and 3-Hydroxy-3-methylpentane-1,5-dioic acid were identified as top 10 up-accumulated metabolites (Figure 2). In contrast, most of the lipids showed down-accumulation under induced cold stress, including N-(2-Hydroxyethyl)eicosapentaenoic acid, LysoPC 19:3, LysoPC 19:0, LysoPC 18:4, LysoPC 19:2, LysoPC 19:2(2n isomer), and LysoPA 16:0 (Figure 3). Further, we identified associated KEGG pathways for DAMs in B144. A significant proportion was associated with metabolic pathways (62.71%), followed by biosynthesis of secondary metabolites (52.94%), biosynthesis of amino acids (23.53%), arginine biosynthesis (17.65%), and purine metabolism (17.65%) (Figure S1A). The association of identified pathways depicted a significant role of these pathways under cold stress.

**Figure 4:**
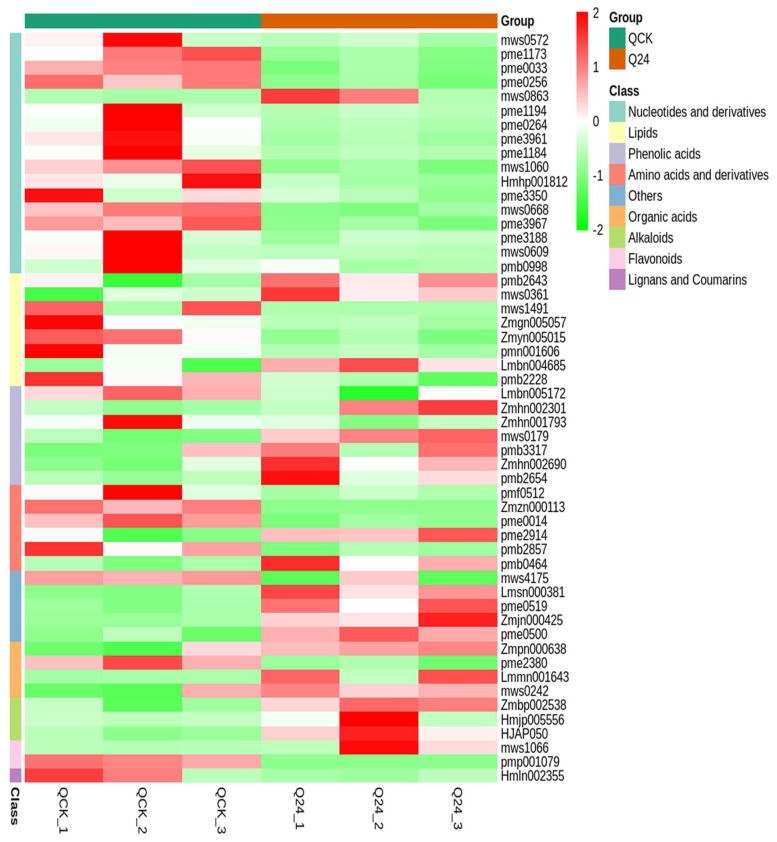
Differential accumulation of metabolites in Q319 (cold tolerant maize inbred line). Heatmap representing accumulation profile of DAMs under control (QCK) and cold-stress (Q24).

**Figure 5:**
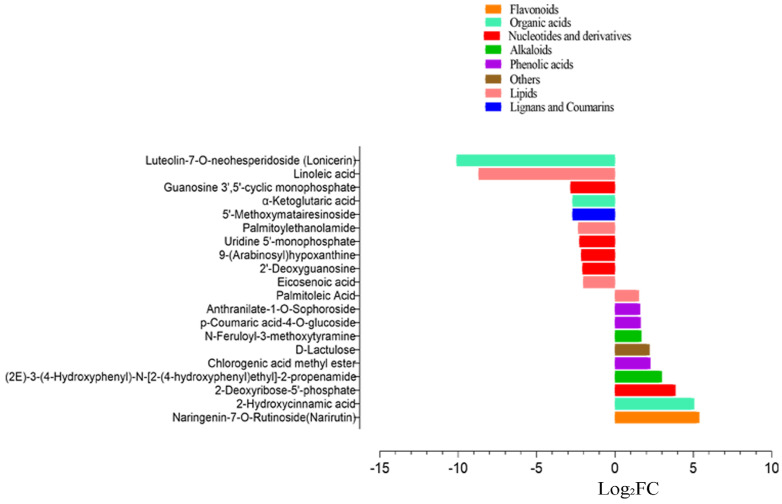
Top metabolites differentially accumulated between QCK and Q24, where the x-axis represents log2FC of ion intensity. * QCK: Q319 control; Q24: Q319 cold stress.

Similarly, cold susceptible inbred line Q319 was also characterized for differential accumulation of metabolites under induced cold stress environment. Fifty-three metabolites were identified as differentially accumulated under cold stress treatment in Q319 (Figure 4 and Table S3). Among them, 22 DAMs were up-accumulated, while 31 DAMs were down-accumulated under cold stress. Naringenin-7-O-Rutinoside(Narirutin), 2-Hydroxycinnamic acid, 2-Deoxyribose-5′-phosphate, (2E)-3-(4-Hydroxyphenyl)-N-[2-(4-hydroxyphenyl)ethyl]-2-propenamide, Chlorogenic acid methyl ester, D-Lactulose, N-Feruloyl-3-methoxytyramine, p-Coumaric acid-4-O-glucoside, Anthranilate-1-O-Sophoroside, and Palmitoleic acid showed up-accumulation in Q319 under cold stress (Figure 5). While, nucleotides and derivatives viz., 2′-Deoxyguanosine, 9-(Arabinosyl)hypoxanthine, Uridine 5’-monophosphate, Guanosine 3′,5′-cyclic monophosphate, Lipids viz., Palmitoylethanolamide, Eicosenoic acid, and Linoleic acid depicted down-accumulation under cold stress (Figure 5). Major KEGG pathways associated with DAMs in Q319 under cold stress were metabolic pathways, biosynthesis of secondary metabolites, purine metabolism, and pyrimidine metabolism (Figure S1B).

### 3.3. Comparative Metabolomics between Cold-Tolerant and Susceptible Maize Inbred Lines

**Figure 6:**
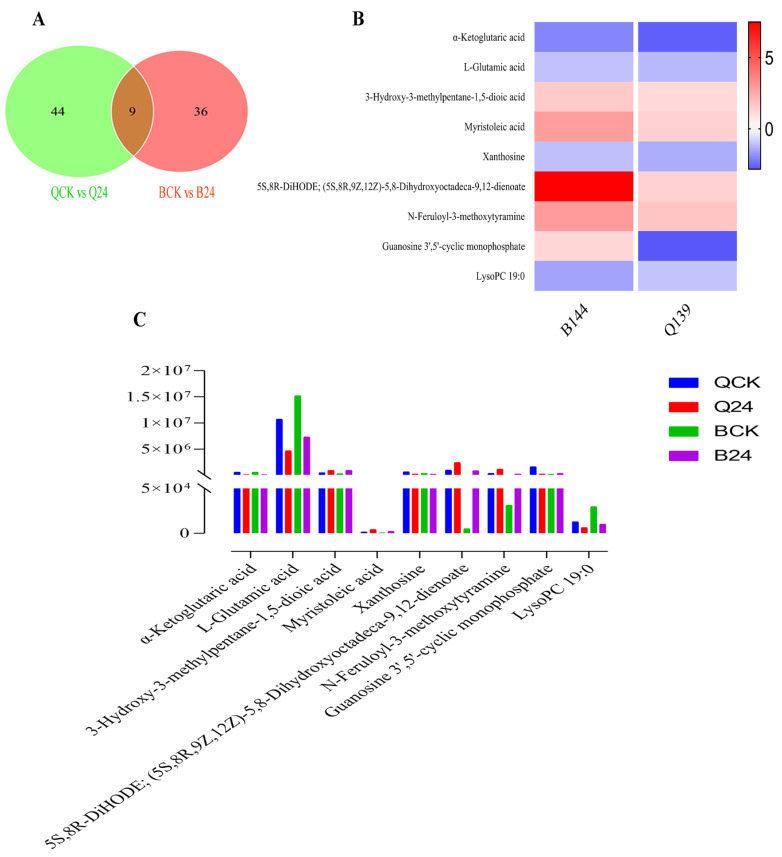
Venn diagram representing common DAMs between different comparisons (**A**) Venn diagram representing common DAMs between BCK vs. B24, and QCK vs. Q24 (**B**) heatmap representing accumulation pattern of conserved DAMs between QCK vs. BCK and Q24 vs. B24 (**C**) Ion intensity of conserved DAMs in QCK, Q24, BCK, and B24. The y axis represents the ion intensity. * QCK: B144 control; B24: B144 cold stress; QCK: Q319 control; Q24: Q319 cold stress.

The response towards stress conditions is generally termed genotype-specific. Therefore, we compared the metabolome of both tolerant and susceptible maize inbred lines and identified nine core cold-responsive metabolites. Figure 6A displays the number of specific and common metabolites differentially accumulated under cold stress between the two genotypes. A detailed description of these DAMs and their corresponding ion intensity has been presented in Table S4.

α-Ketoglutaric acid, L-Glutamic acid, 3-Hydroxy-3-methylpentane-1,5-dioic acid, Myristoleic acid, Xanthosine, 5S,8R-DiHODE; (5S,8R,9Z,12Z)-5,8-Dihydroxyoctadeca-9,12-dienoate, N-Feruloyl-3-methoxytyramine, and LysoPC 19:0 showed similar accumulation pattern (up/down-accumulated) in both inbred lines B144 and Q319 (Figure 6B). However, their relative accumulation was significantly different between the two lines (Figure 6C). α-Ketoglutaric acid, L-Glutamic acid, Xanthosine, and LysoPC 19:0 showed down-accumulation in both inbred lines, while 3-Hydroxy-3-methylpentane-1,5-dioic acid, Myristoleic acid, 5S,8R-DiHODE; (5S,8R,9Z,12Z)-5,8-Dihydroxyoctadeca-9,12-dienoate, and N-Feruloyl-3-methoxytyramine depicted up-accumulation.

Although most conserved DAMs depicted similar accumulation patterns in both inbred lines, their accumulation was significantly different in both inbred lines under different treatments. For instance, L-Glutamic acid showed higher accumulation under control (with significant differences in both inbred lines), while a significant reduction in accumulation was observed under cold treatment (Figure 6C). Histidine metabolism, arginine biosynthesis, carbon metabolism, metabolic pathways, Citrate cycle (TCA cycle), lysine biosynthesis, biosynthesis of amino acids, and biosynthesis of secondary metabolites were major KEGG pathways associated with these DAMs (Figure S1 and Table S4). The similar distribution pattern of the above-mentioned conserved DAMs suggests a prophylactic response in both cold tolerant and susceptible inbred lines. Besides, Guanosine 3′,5′-cyclic monophosphate showed up-accumulation in B144 (BCK vs. B24), while it showed down-accumulation in Q319 (QCK vs. Q24), which could be a good biomarker to screen tolerant and sensitive genotypes.

## 4. Discussion

Cold stress at the early growth stages is critical for thermophilic plant species, causing significant losses during progressive growth stages [39,40]. Several reports suggested severe damage in maize crop under cold stress at early growth stages [16,41,42,43]. The maize crop is generally grown early in the season, under a temperate climate, to escape heat stress at later growth stages [5]. However, early sown maize crop is exposed to chilling stress (0–15 °C). A considerable variation is present in maize germplasm in response to cold stress [44,45,46]. Therefore, it is critical to investigate and understand genetic regulators and metabolic responses resulting from chilling stress in cold-tolerance and cold-succeptible maize accessions. Hereby, we evaluated two maize inbred lines B144 (Cold tolerant) and Q319 (Cold susceptible) for their metabolic profile under induced cold stress condition.

Abiotic stresses impact plants by inducing physiological, molecular, and biochemical changes disturbing growth and development. Disorganized cell membrane, altered osmotic stress, denatured proteins, and increased reactive oxygen species (ROS) could result in oxidative damage are among primary responses [47]. The sessile nature of plants helps develop complex responsive mechanisms, including multiple pathways under stress conditions [10,48]. Regulation pathways under cold stress may vary from species to species [49]. For instance, Hao et al. suggested enrichment of flavonoid biosynthesis, phagosome, plant hormone signal transduction, and fructose at the onset of cold stress in tea plants. In contrast, sugar metabolism, alanine biosynthesis, and the aspartate and glutamate metabolism were found enriched during later stage of cold induction [50]. On the other hand, Mata et al. identified downregulation in ethylene signaling under cold stress in tomato [51]. Moreover, Rubio et al. emphasized that upregulation of ABA synthesis genes plays a crucial role in the grapevine to induce cold hardiness [52]. Based on previous reports, it can be inferred that regulatory genes have varying functions in different plant species with a considerable variation in signal transduction pathways and metabolism while increasing complexity of cold tolerance mechanisms [49]. C-repeat binding factors are core regulators of cold-response genes and transcription factors such as protein kinases, MADS, WRKY, NAC, and TRAF [53,54], while secondary metabolites such as lignin, anthocyanin, and amino acids protect cellular components from cold-induced damages [55,56,57]. The metabolic profiles of B144 and Q319 suggested differential accumulation of metabolites compared with their corresponding control. Cold-stress mediated response in two inbred maize lines was identified as conserved DAMs between BCK vs. B24 and QCK vs. Q24. A similar approach has been adopted by Yu et al. [27]. Among nine conserved DAMs between BCK vs. B24 and QCK vs. Q24, only Guanosine 3′,5′-cyclic monophosphate showed differential accumulation between BCK vs. B24, and QCK vs. Q24 (Up-accumulated in B144 and down-accumulated in Q319). Guanosine 3′,5′-cyclic monophosphate has been characterized as an important signal compound meditating multiple adaptive responses under environmental stresses [58]. Guanosine 3′,5′-cyclic monophosphate has been previously reported to mediate gibberellic acid-induced chilling tolerance in peach [59] and other plants [60]. Therefore, we interpreted up-accumulation of Guanosine 3′,5′-cyclic monophosphate plays a crucial role in developing cold tolerance in B144.

Genotype-specific cold stress-mediated responses as DAMs were identified by comparing metabolome changes under cold stress and control conditions in two inbred maize lines. B144 depicted up-accumulation of several metabolites, viz., flavonoids, lipids, amino acids, alkaloids, and phenolic acids under induced cold stress. Several studies have suggested genotype-dependent regulatory responses under abiotic stresses [61,62,63,64]. Survival strategies under a stress environment highly depend on vegetative growth regulation and hormonal distribution [65]. Auxins, an essential class of regulators, transportation to stem and roots is mediated by protein carriers [27,66,67,68]. However, the role of secondary metabolites in auxin transportation is still unknown. Previous reports also emphasized the role of flavonoids in regulating indole acetic acid (IAA), a key component in auxin biosynthesis [68]. In contrast, a previous report suggested that a higher accumulation of flavonoids can disturb auxin transport [65]. Interestingly, Quercetin-3-O-(2″′-p-coumaroyl) sophoroside-7-O-glucoside depicted up-accumulation in B144 under cold stress. A previous report suggested β-linked glucosides play a crucial role during abiotic stress recovery [69]. Remaining up-accumulated flavonoids, viz., Phloretin [65], Phloretin-2′-O-glucoside (Phlorizin) [70,71], Chrysoeriol-7-O-(6″-acetyl)glucoside [72], Chrysoeriol-5-O-glucoside [73], and Quercetin-3-O-rutinoside-7-O-rhamnoside [74] have been reported for their effective role in plant development under stress environment. Similarly, in Q319, we identified up-accumulation of flavonoids, viz., Naringenin-7-O-Rutinoside and Luteolin-7-O-neohesperidoside (Lonicerin). Differential accumulation of these flavonoids under cold stress in cold-tolerant B144 and cold-susceptible Q319 suggested a response towards cold stress.

Amino acids, as osmoprotectants, play a critical role under temperature stress conditions in plants [75]. Protein oxidation by ROS is considered irreversible; however, proteins with sulfur-containing amino acids are an exception [76]. A previous report [10] addressing the cold tolerance in maize at the seedling stage suggested upregulation of genes encoding β-alanine aminotransferase and glutamate decarboxylase involved in the biosynthesis of β-alanine betaine and γ-aminobutyric acid, which play a crucial role in plant development under environmental stresses [77,78,79]. Our study depicted up-accumulation of 3-Hydroxy-3-methylpentane-1,5-dioic acid, L-Lysine-Butanoic Acid, L-Leucyl-L-phenylalanine, L-Alanyl-L-Phenylalanine, L-Glutamine, L-Lysine, L-Valyl-L-Phenylalanine, and L-Aspartyl-L-Phenylalanine in B144 under cold stress. Matysiak et al. reported positive growth regulation of maize crop under exogenous application of L-Arginine [75]. 3-Hydroxy-3-methylpentane-1,5-dioic acid was previously identified, supplementing Arginine biosynthesis, as a cold-responsive metabolite in Ipomoea batata [80]. L-Lysine [81,82], L-phenylalanine [83], and L-Glutamine [84] have been previously reported for their essential role in growth regulatory responses in plants. L-glutamate is considered a precursor of Glutamine, Proline, and Arginine [85]. Glutamine and Arginine synthesis pathways have been reported as essential regulators for plant growth and development under cold stress [86]. Furthermore, Nitric-oxide synthase, arginase, and arginine decarboxylase play a critical role in arginine metabolism affecting the proline and polyamines [86].

Phenolics are important natural metabolites in plant defense responses against stress through the formation of metallic complexes by scavenging the ROS while inhibiting oxidative enzymes [87,88]. Previous reports emphasized efficient regulation of phenolics viz., salicylic acid, ortho-hydroxycinnamic acid, abscisic acid, and jasmonic acid under cold stress in crop plants [87,89,90,91,92]. Abscisic acid is a known regulator under cold stress providing interaction between ABA-dependent and ABA-independent pathways [93]. Moreover, Chen et al. [94] provided evidence for improved cold tolerance in maize under exogenous application of abscisic acid. A recent study concerning cold tolerance in maize depicted up-accumulation of ZEP, NCED, β-carotene isomerase β-carotene 3-hydroxylase, and ABA 8′-hydroxylase under cold stress [10]. Our results depicted up-accumulation of Feruloyltartaric acid (Fertaric acid), and Sinapyl alcohol under stress conditions in B144. Cell wall stability is important for plants to survive cold stress, and Sinapyl alcohol has been previously identified to support cell was stability under cold stress [95]. The phenolics mentioned above have also been reported for their active role in coping with stress conditions in plants [96,97,98,99].

## 5. Conclusions

Our study, utilizing a widely targeted metabolomics approach, emphasized the differential accumulation of metabolites in tolerant and susceptible maize inbred lines viz., B144, and Q319, respectively. The identification of key metabolites viz., Guanosine 3′,5′-cyclic monophosphate, Quercetin-3-O-(2″′-p-coumaroyl)sophoroside-7-O-glucoside, Phloretin, Phloretin-2′-O-glucoside, Naringenin-7-O-Rutinoside, L-Lysine, L-phenylalanine, L-Glutamine, Sinapyl alcohol, and Feruloyltartaric acid, as cold-mediated responses, laid the foundation for metabolome changes associated with cold tolerance in maize. The study provides a theoretical basis for further exploration of cold-mediated metabolic responses and their regulation in maize.

## Data Availability

The data used are available within the text and its Supplementary Materials.

## Supplementary Materials

The following supporting information can be downloaded at: https://www.mdpi.com/article/10.3390/life12020282/s1, Table S1: Metabolome dataset for cold mediated responses in two inbred maize lines viz., B144 and Q319 under cold stress and control conditions; Table S2: Differential accumulation of metabolites in B144 maize inbred line under control and cold-stress conditions; Table S3: Differential accumulation of metabolites in Q319 maize inbred line under control and cold-stress conditions; Table S4: Core metabolites identified as a response to cold stress; Figure S1: KEGG pathways associated with DAMs identified in B144 (BCK vs. B24) and Q319 (QCK vs. Q24).
