# Supplementary figures and images for: Metabolic Insight into Cold Stress Response in Two Contrasting Maize Lines
Source: Life (Basel). 2022 Feb 14;12(2):282. doi: 10.3390/life12020282 (PMC8875087; doi:10.3390/life12020282)

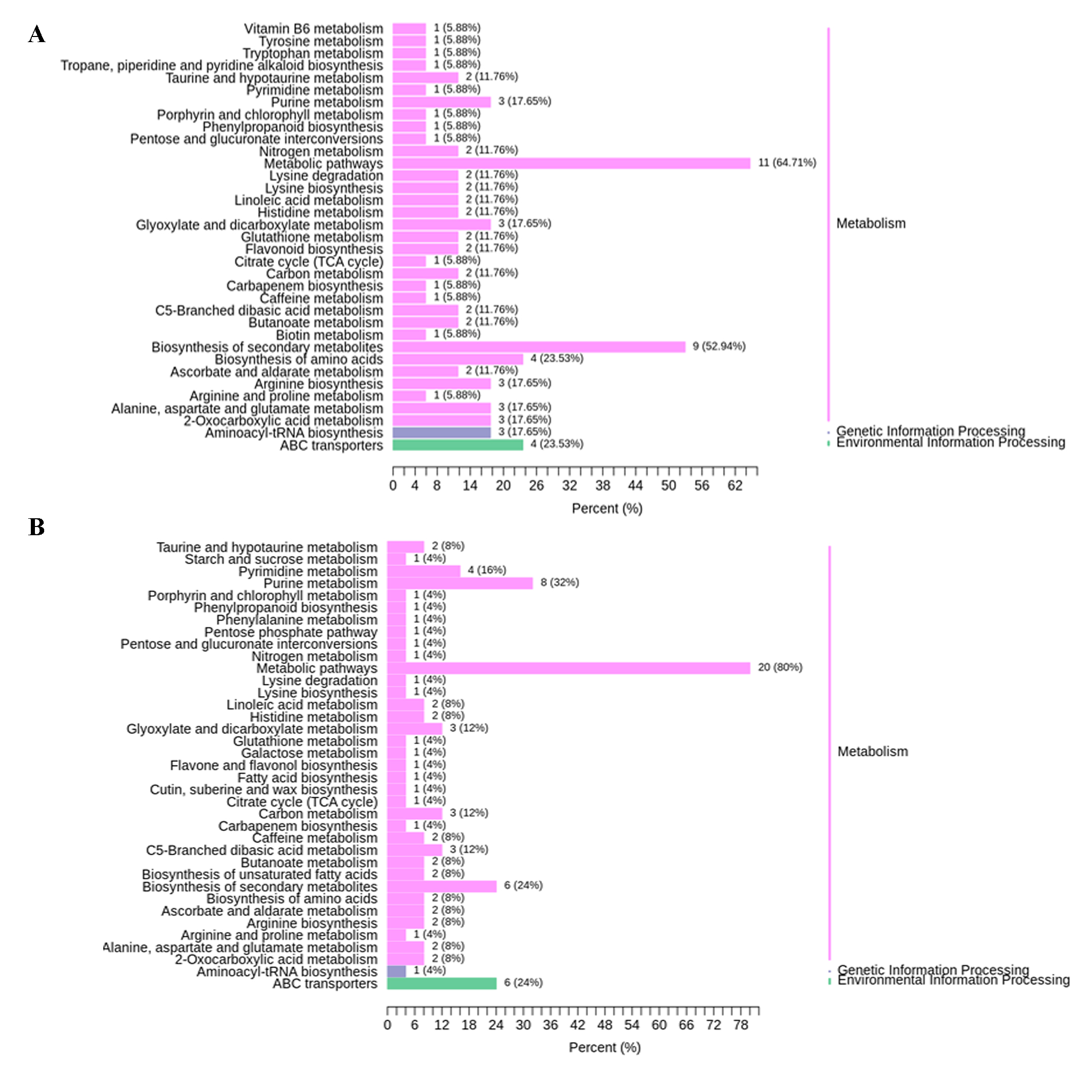

Supplement: Supplementary file 1 [file life-12-00282-s001.zip › life-1545905-supplementary/Supplementary Information/Figure S1.JPEG]
